# The Role of Digital Rectal Examination for Early Detection of Significant Prostate Cancer in the Era of Magnetic Resonance Imaging

**DOI:** 10.3390/life14111359

**Published:** 2024-10-23

**Authors:** Juan Morote, Nahuel Paesano, Natàlia Picola, Jesús Muñoz-Rodriguez, Xavier Ruiz-Plazas, Marta V. Muñoz-Rivero, Ana Celma, Gemma García-de Manuel, Berta Miró, José M. Abascal, Pol Servian, Olga Méndez, Enrique Trilla

**Affiliations:** 1 Department of Urology, Vall d’Hebron University Hospital, 08035 Barcelona, Spain; anna.celma@vallhebron.cat (A.C.); enrique.trilla@vallhebron.cat (E.T.); 2 Department of Surgery, Universitat Autònoma de Barcelona, 08193 Bellaterra, Spain; nahuel.paesano@uab.cat; 3 Urology Research Unit, Vall d´Hebron Research Institute, 08035 Barcelona, Spain; olga.mendez@vhir.org; 4 Clínica Creu Blanca, 08034 Barcelona, Spain; 5 Department of Urology, Hospital Universitari de Bellvitge, 08907 Hospitalet de Llobregat, Spain; npicola@bellvitgehospital.cat; 6 Department of Urology, Hospital Universitari Parc Taulí, 08208 Sabadell, Spain; jmunoz@tauli.cat; 7 Department of Urology, Hospital Universitari Joan XXIII, 43005 Tarragona, Spain; xruiz.tarragona.ics@gencat.cat; 8 Department of Urology, Hospital Univeritari Arnau de Vilanova, 25198 Lleida, Spain; mvmunoz.lleida.isc@gencat.cat; 9 Department of Urology, Hospital Universitari Josep Trueta, 17007 Girona, Spain; gemmagarcia.girona.ics@gencat.cat; 10 Statistics Unit, Vall d´Hebron Research Institute, 08035 Barcelona, Spain; berta.miro@vhir.org; 11 Department of Urology, Parc de Salut Mar, 08003 Barcelona, Spain; jabascal@psmar.cat; 12 Department of Medicine and Health Science, Universitat Pompeu Fabra, 08003 Barcelona, Spain; 13 Department of Urology, Hospital Universitari Germans Trias i Pujol, 08916 Badalona, Spain; pservian.germanstrias@gencat.cat

**Keywords:** prostate cancer, early detection, digital rectal examination

## Abstract

The role of digital rectal examination (DRE) in the early detection of significant prostate cancer (PCa) is being questioned in the era of magnetic resonance imaging (MRI). However, some men with suspected PCa may still be identified solely through DRE, even with low serum prostate-specific antigen (PSA) levels. Additionally, most predictive models designed to improve significant PCa diagnostic pathways incorporate DRE findings. We assessed the role of DRE among 5005 men with serum PSA levels > 3.0 ng/mL and/or suspicious DRE findings, who underwent pre-biopsy MRI and targeted and/or systematic biopsies, as part of the significant PCa opportunistic screening program in Catalonia (Spain) between 2016 and 2023. Significant PCa, defined as grade group > 2, was detected in 2097 men (41.9%). Suspicion of PCa was based solely on DRE in 206 cases (4.1%) with significant PCa detected in 50 of them (2.4%). Two pathways using the Barcelona predictive models, before and after MRI, with and without DRE findings showed specificities of 52.8 and 38.7%, respectively (*p* < 0.001), after fixing sensitivity at 90%. Prostate biopsy was avoided in 35.1 and 26.7%, respectively (*p* < 0.001), while its efficacy increased from 52.8 to 58%. We conclude that DRE improved the effectiveness of an opportunistic significant PCa-screening program.

## 1. Introduction

Digital rectal examination (DRE) was the only method to screen men suspected of having prostate cancer (PCa) until the discovery and spread of serum prostate-specific antigen (PSA) in the early 1980s [1]. In 1994, serum PSA testing was approved by the United States Food and Drug Administration to screen men suspected of having PCa, being used jointly with DRE in opportunistic screening programs [2]. However, population-based screening programs have utilized serum PSA alone, lowering the thresholds for detection [3,4]. PCa screening has been controversial for a long time as a reduction in PCa-specific mortality was evident. The European Randomised Study of Screening for Prostate Cancer initiative demonstrated, in 2009, a 20% decrease in PCa mortality after seven years of follow-up [4]. This drop in the specific mortality resulted from the early detection and appropriate treatment of significant PCa tumors with a grade group two or higher, which increased up to 29% after a 22-year follow-up in the Göteborg Randomised Population-Based Prostate Cancer Screening Trial [5]. The new paradigm of PCa screening therefore focuses on the early detection of significant PCa. The widespread use of magnetic resonance imaging (MRI) and targeted biopsies of suspicious lesions has facilitated an increase in significant PCa detection with a reduction in unnecessary prostate biopsies and the over-detection of insignificant PCa, although uncertain scenarios remain [6]. 

The European Union currently recommends population-based PCa screening and encourages member states to assess the feasibility and effectiveness of risk-organized screening programs [7]. Such programs will identify men suspected of having PCa within the age-appropriate healthy men that are invited for serum PSA testing, and those suspected of having PCa will undergo MRI and prostate biopsy of prostate imaging–reporting and data system (PI-RADS) lesions > 3 [8]. The European Association of Urology also encourages the design of risk-stratifying diagnostic pathways based on predictive models to improve the efficiency of significant PCa screening [9]. Some of these pathways have shown significant reductions in MRI demand, unnecessary prostate biopsies, and the over-detection of insignificant PCa [10,11,12]. We highlight that most predictive models, designed for the stratification of men suspected of having PCa before and after MRI, incorporate DRE findings, suspicious vs. normal, and DRE-derived prostate volume categories since these variables are independent predictors of significant PCa [13,14,15]. Currently, opportunistic significant PCa screening programs are frequently implemented in areas where population-based screening has not been introduced. In these settings, physicians and candidates jointly agree to screen PCa, with serum PSA testing and DRE being offered [16]. However, the usefulness of DRE alongside serum PSA testing for screening men suspected of having PCa is controversial [17,18]. Two recent meta-analyses have advised against DRE in the early detection of PCa. However, both meta-analyses included studies in which pre-biopsy MRI was not conducted [19,20]. Additionally, DRE in routine practice is increasingly being rejected, even by urologists themselves [21,22,23,24,25].

We hypothesize that DRE is useful for significant PCa detection in an opportunistic screening program developed in the era of MRI. Our objectives were (i) to verify if DRE remains an independent predictor of significant PCa, (ii) to assess the rate of significant PCa detected solely through a suspicious DRE, and (iii) to compare the effectiveness of two diagnostic pathways including (or not) DRE findings in the predictive models used to stratify men suspected of having PCa before and after MRI.

## 2. Materials and Methods

### 2.1. Design, Setting, and Participants

This is a cohort study, in which a retrospective review of DRE’s usefulness for sPCa detection was conducted in a population prospectively recruited for the development and validation of the significant PCa Barcelona predictive models (BCN-PMs). A cohort of 5005 men suspected of having PCa, due to a serum PSA level > 3.0 ng/mL and/or suspicious DRE findings, who underwent pre-biopsy MRI and targeted and/or systematic biopsies as part of the significant PCa opportunistic screening program of Catalonia (Spain) between 2016 and 2023 was analyzed [15,26]. This program is conducted by urologists who perform DRE in the primary care setting. Thereafter, men suspected of having PCa were referred to local participant centers for MRI scanning and prostate biopsy if indicated.

This study was conducted in accordance with the Declaration of Helsinki and approved by the Institutional Review Board of Vall d´Hebron Hospital (PRAG-02/2021) on 12 February 2021. Informed consent was obtained from all subjects involved in the study.

### 2.2. Data Analysis

Anonymized datasets were provided by participant centers. The independence of DRE in predicting significant PCa was analyzed. The rate of significant PCa detected solely through suspicious DRE findings was estimated. Rates of significant PCa detection, the avoidance of MRI exams and prostate biopsies, and prostate biopsy efficacy were compared between two diagnostic pathways using BCN-PMs with and without DRE findings.

### 2.3. Significant Prostate Cancer Diagnostic Pathway

After PCa suspicion, an MRI was performed in a 1.5 or 3 Tesla scanner with a stan-dard surface phased-array coil at reference centers within a month of PCa suspicion. The overall acquisition protocol included T2-weighted imaging, diffusion-weighted imaging, and dynamic contrast-enhanced imaging, according to the guidelines of the European Society of Urogenital Radiology [27]. Expert radiologists analyzed images using the PI-RADS v2.0 before and v2.1 after 2019 in each participant center [28,29].

Prostate biopsies were conducted by expert operators in each participant center after lesions segmentation. Men with PI-RADS 3 to 5 underwent 2- to 4-core transrectal ultrasound-targeted biopsies for all lesions and 12-core systematic biopsies. Men selected for the prostate biopsy with PI-RADS < 3 underwent a 12-core systematic biopsy [16].

Biopsy samples were sent separately to local pathology departments, where expert Uro-pathologists assigned the International Society of Urologic Pathology grade group when PCa was detected. Significant PCa was defined when the grade group was 2 or higher [30].

### 2.4. Variables in the Study

Age at the time of prostate biopsy (years), PCa family history (present vs. absent), type of biopsy (initial vs. repeated), serum PSA (ng/mL), DRE (normal vs. suspicious), DRE-prostate volume category (small, medium, and large) and PI-RADS score (1 to 5) were recorded as variables. The outcome variables were PCa, significant PCa, insignificant PCa (no vs. yes), and grade group of PCa (1 to 5).

### 2.5. Statistical Analysis

Data were prospectively collected and reported according to the Standards of Repor-ting for MRI-targeted Biopsy Studies to describe the study population [31]. The reported anonymized datasets were harmonized at the coordinating center. Quantitative variables were expressed as medians and interquartile ranges (IQR = 25 ptile–75 ptile) and qualitative variables were expressed as percentages. Comparisons between quantitative variables were performed using the Mann–Whitney U test and relative risks (RR) estimated with their 95% confidence intervals (CI). Kruskal–Wallis tests were used when more than two groups were compared. Percentages were compared with the Chi-square test. A stepwise logistic regression was used to search for independent predictive variables of significant PCa. Odds ratios (ORs) and their 95% CI were estimated. BCN-like PMs 1 and 2, based on the same predictive variables as the BCN-PMs, were developed without DRE findings, and the likelihood of significant PCa was estimated. Significant differences were identified as *p*-value < 0.05. The data were analyzed using the Statistical Package for the Social Sciences (version 29.0; IBM Corp., Armonk, NY, USA).

## 3. Results

### 3.1. Characteristics of Study Population

The overall characteristics of the study population are summarized in Table 1. Additionally, age ranged between 37 and 85, with 3016 (60.3%) and 1989 (39.7%) men younger and older than 70 years, respectively. The serum PSA ranged from 0.7 and 112 ng/mL; in 206 men (4.1%), it was ≤3.0 ng/mL; in 3419 men, it was between 3.1 and 10.0 ng/mL (68.3%); and in 1380 men, it was >10 ng/mL (27.6%). The MRI-derived prostate volume was ≤50 mL in 2245 (44.9%) and >50 mL in 2759 (55.1%) men. A negative MRI (PI-RADS < 3) was observed in 749 men (15%) while PI-RADS scores ranged from 3 to 5 in 4256 (85%). Significant PCa was detected in 2097 men (41.9%). The characteristics of the population with normal DRE were significantly different than those observed in men with suspicious DRE.

### 3.2. Independence of DRE for Significant Prostate Cancer Detection

A stepwise logistic regression analysis was carried out to find the independent predictors of significant PCa (Table 2). Age, first degree of PCa family history, serum PSA level, suspicious DRE, and PI-RADS score were directly related to significant PCa detection, while a previous negative prostate biopsy and prostate volume were negatively related to significant PCa detection. DRE was the second most powerful independent predictor of significant PCa, behind the PI-RADS score.

**Figure 1:**
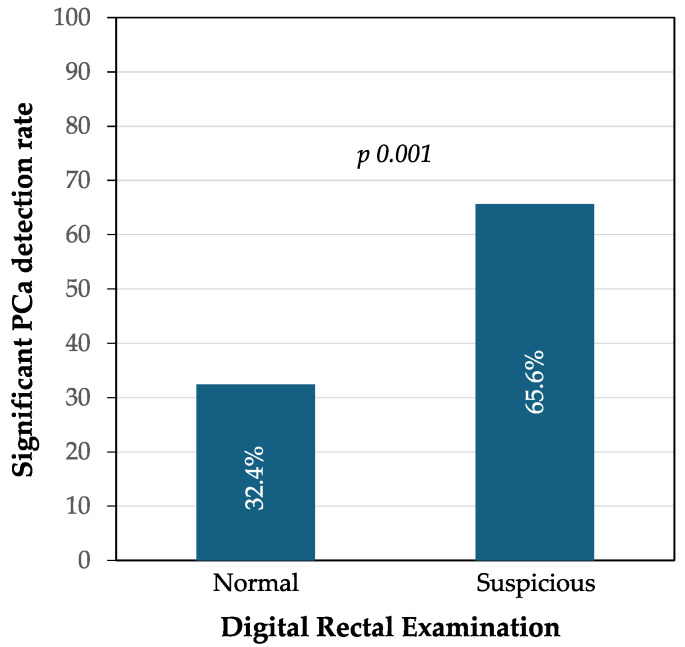
Significant PCa detection rate according to DRE findings.

Among 1427 men with suspicious DRE, 936 (65.6%) cases of significant PCa were detected compared to 1116 among 3578 (32.4%) with normal DRE (*p* < 0.001). The OR was 3.969 (95% CI 3.486–5.518) and the RR was 1.963 (95% CI 1.821–2.116) for suspicious DRE and significant PCa detection, as shown in Figure 1.

Among 206 men with serum PSA levels below the detection threshold, significant PCa was detected in 8 of 40 (20%) with serum PSA levels of 0.7 to 1.0 ng/mL, 12 of 59 with serum PSA levels of 1.1 to 2.0 ng/mL, *p* = 0.623, and 30 of 107 (28%) with serum PSA levels of 2.1 to 3.0 ng/mL, *p* = 0.011 regarding the other two subsets.

### 3.3. Significant Prostate Cancer Detection Solely Through Suspicious DRE

Among the subset of 206 men in whom the suspicion of PCa was solely established through an abnormal DRE finding with serum PSA levels up to 3.0 ng/mL (4.2%), significant PCa was diagnosed in 50 of them (24.3%). They represented 2.4% of 2097 significant PCa cases detected and an overall detection rate of 1% (in reference to the 5005 participants).

### 3.4. Effect of DRE Findings for Improving the Efficacy of the Barcelona Diagnostic Pathway

**Figure 2:**
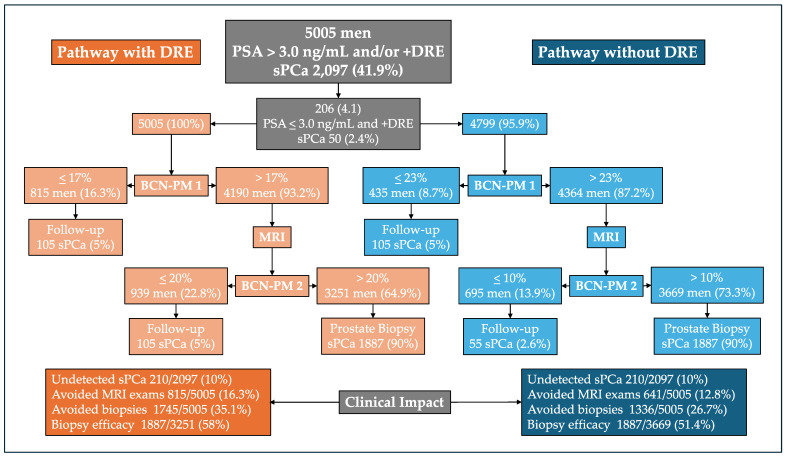
Decision tree analysis of significant PCa diagnostic pathway, stratifying men for MRI and prostate biopsy by the BCN-PMs 1 and 2 (pathway with DRE), and BCN-like PMs 1 and 2 without including the DRE-derived variables (pathway without DRE).

The behavior of the Barcelona diagnostic pathway, based on stratifications conducted by the BCN-PMs 1 and 2 before and after MRI and the BCN-like PMs 1 and 2 (developed without DRE findings), among the 5005 men suspected of having PCa from the opportunistic significant PCa screening program in Catalonia is presented in Figure 2. Initially, since 206 men were suspected of having PCa due only to a suspicious DRE, the diagnostic pathway without DRE was analyzed in 4799 men, while that using DRE was analyzed in 5005 men. The thresholds of the predictive models were selected to obtain an overall 90% sensitivity for significant PCa detection in both pathways. The overall specificity was 52.8% in the pathway using DRE findings and 38.7% in the one that did not use them, *p* < 0.001. In terms of the clinical impact, the rate of undetected significant PCa was 10% in both pathways, while 16.3 and 12.8% of MRI exams were avoided, respectively, *p* < 0.001. The rates of avoided prostate biopsies were 35.1 and 26.7%, respectively, *p* < 0.001. The efficacy of prostate biopsy (positive predictive value) in both pathways was 58.0% and 52.8%, respectively, *p* < 0.001. The expression “Follow up” in men in whom MRI and prostate biopsy will be avoided means that sPCa will be detected when a prostate biopsy is conducted in the future if PCa suspicion remains.

### 3.5. Aggressiveness of Significant PCa Detected in Men Suspected of Having PCa Solely Identified with DRE

The grade group of the 50 significant PCa cases detected when serum PSA levels were up to 3.0 ng/mL were as follows: grade 2 in 26 cases (52%), grade 3 in 15 (30%), grade 4 in 8 (16%), and grade 5 in 5 cases (10%). This grade group rate distribution was similar to that observed within the 2047 significant PCa cases detected in men with serum PSA levels above 3.0 ng/mL, 45.5, 30.4, 13.7, and 10.5%, respectively, *p* = 0.198.

## 4. Discussion

The current study reports that DRE remains an independent predictor of significant PCa detection in an opportunistic significant PCa screening program developed in the era of MRI. The likelihood of significant PCa in men with a suspicious DRE was nearly double that of men with normal DRE findings. Men suspected of having PCa based solely on a suspicious DRE constituted 4.2% of participants, and significant PCa was detected in 2.4% of all significant PCa cases. The overall detection rate of significant PCa, based solely on a suspicious DRE, among the 5005 participants in this study was 1%. An increase in significant PCa detection from 20 to 28% was observed in men with serum PSA levels between 2.1 and 3.0 ng/mL compared to those with lower serum PSA levels. The diagnostic pathway for improving significant PCa screening, using stratifications before and after MRI based on BCN-PMs 1 and 2, as compared to the BCN like-PMs, which did not include DRE findings, reduced MRI demand from 12.8 to 16.3%, reduced unnecessary biopsies from 26.7 to 35.1%, and improved the efficacy of prostate biopsy from 51.4 to 58%. Finally, the aggressiveness of significant PCa detected solely through suspicious DRE findings, based on the grade group distribution, was similar to that observed in cases detected when serum PSA levels were above 3.0 ng/mL.

The 28.5% incidence of suspicious DRE findings in the present series was similar to that reported in other series from opportunistic screening programs. The incidence of suspicious DRE findings varies notably according to the age of participants, percentage of previous negative prostate biopsies, type of screening program, and screening round [2,13,14,19,21,31,32,33,34,35,36]. A common criticism of DRE is its high inter-examiner variability, even when conducted by expert urologists [25]. However, an important point in favor of the current use of DRE to predict the risk of significant PCa is that it remained an independent predictive factor when the PI-RADS score was included in the logistic regression analysis [10,11,12,13,14,15]. As in other studies, DRE almost doubled the risk of significant PCa compared to men with normal DRE results [2,3,20,37,38,39,40,41,42,43].

From a clinical point of view, the main reason for recommending DRE alongside serum PSA testing is to increase significant PCa detection in men with suspicious DRE findings and low serum PSA levels. In our series of 5005 men who underwent prostate biopsy after an MRI, we identified 50 men with significant PCa solely detected through a suspicious DRE finding. This represented a 1% increase in the overall significant PCa detection rate, which was similar to that reported in the recent meta-analysis by Matsukawa et al. [20]. These authors concluded that DRE does not add value to serum PSA testing. In our series, the 50 significant additional PCa cases detected in men with serum PSA levels up to 3.0 ng/mL represented a likelihood of 24.3%, which is higher than that reported in men with PI-RADS 3 [44,45]. On the other hand, these cases represented 2.4% of the 2097 overall significant PCa cases detected, although the overall increase in significant PCa detection was also 1%. In 2018, Halpern et al. analyzed 35,350 men participating in the screening arm of the Prostate, Lung, Colorectal, and Ovarian cancer screening trial, in which DRE and serum PSA testing were conducted jointly [28]. The incidence of significant PCa increased from 13.7 to 23% when suspicious DRE was found in men with serum PSA levels above 3.0 ng/mL. Interestingly, the authors reported an increase from 3.5 to 6.5% in significant PCa detection when serum PSA levels ranged between 2.1 and 3.0 ng/mL, and from 0.7 to 1% when serum PSA levels were lower than 2.1 ng/mL [35]. These data correlate with our findings, suggesting that suspicious DRE increases the detection of significant PCa in the overall population. We have observed that suspicious DRE increased the risk of significant PCa from 32.4 to 65.6%. In men with serum PSA levels up to 3.0 ng/mL and suspicious DRE findings, we observed an increase in significant PCa from 20% when serum PSA ranged from 0.7 and 2.0 ng/mL, and 28% when it ranged from 2.1 to 3.0 ng/mL. Gosselar et al. analyzed the positive predictive value of DRE in the first three screening rounds at the Rotterdam section of the European Randomised Screening Prostate Cancer trial in 5040 prostate biopsies conducted in men referred due to a serum PSA level of 3.0 ng/mL or higher. PCa was detected in 48.6% of men with suspicious DRE compared to 22.4% without in the first round, with 71% of these being significant PCa; 29.9% compared to 17.1% during the second round, with 68.8% being significant PCa; and 21.2% vs. 18.2% during the third round, with 85.7% being significant PCa. The authors noted that the prediction power of DRE for detecting significant PCa in men with elevated serum PSA was maintained over twelve years in a population-based PCa screening program [34]. Our finding of similar aggressiveness of significant PCa in men with serum PSA levels below and above 3.0 ng/mL has not been previously reported and suggests that omitting DRE in population-based screening programs may delay detection until subsequent rounds, when serum PSA rises above 3.0 ng, without an increase in aggressiveness.

Risk-stratified population-based significant PCa screening programs are currently recommended by the European Union through an invitation for serum PSA testing from health authorities, similar to mammography or fecal occult blood for breast or colorectal cancers [7,8,9]. This is important since population-based screening programs reduce the specific mortality of PCa significantly more effectively than opportunistic screening programs [46]. However, it must be recognized that the detection of some significant PCa is delayed when DRE is utilized [20]. We have demonstrated that DRE improves the effectiveness of the diagnostic pathway based on stratifications of men suspected of having PCa, both before and after MRI with BCN-PMs [11,12]. Therefore, the question is not whether to use DRE in the early detection of significant PCa, but rather when to conduct it in the diagnostic pathway of significant PCa. An expert DRE should be performed before MRI to stratify men suspected of PCa and select appropriate candidates for MRI. A suspicious DRE finding is an important predictor of significant PCa and is always included in predictive models, both before and after MRI, to improve the efficacy of screening. We also highlight that prostate inflammation can be suspected when pain occurs under digital pressure on the posterior gland surface. Inflammation is an important cause of non-PCa elevation of serum PSA levels and a reason for delaying and perhaps avoiding prostate biopsy [47,48]. Other novel markers can be useful [49]. Finally, DRE can identify the T-stage of potentially detected PCa, avoiding the inconvenience of conducting it after a prostate biopsy [16]. Effective educational methods for non-experts in performing DRE are needed in significant PCa screening programs [50].

This study has limitations. It was designed retrospectively, and the variability among participating centers and urologists conducting DRE may have introduced bias into the results. The definition of significant PCa in prostate biopsies may not fully represent the pathology of the entire prostate gland. Additionally, our study was conducted within an opportunistic screening program, which typically targets a different profile of participants compared to population-based screening programs. Therefore, the results may be difficult to generalize. The strengths of our study include the large sample size, the multicentric design, and the involvement of experienced urologists who were familiar with the primary healthcare system and the diagnostic pathway.

## 5. Conclusions

DRE remains a powerful predictor of significant PCa in the MRI era. Suspicious DRE findings double the overall risk of significant PCa in prostate biopsies. DRE enhances the identification of men suspected of having PCa, allowing the detection of 2.4% of significant PCa cases. The diagnostic pathway for significant PCa, including DRE in predictive models applied before and after MRI, reduced MRI demand and unnecessary prostate biopsies compared to models without DRE, thereby increasing overall efficacy.

## Figures and Tables

**Table 1:** Characteristics of study population.

Characteristic	Measurement	−DRE	+DRE	*p*-Value
Number of men	5005	3578 (71.5)	1427 (28.5)	0.001
Median age, years (IQR)	68 (62–74)	67 (61–73)	69 (64–76)	0.001
Median serum PSA, ng/mL (IQR)	7.0 (5.0–10.8)	9.8 (5.0–9.6)	21 (9.8–9.9)	0.001
Repeated biopsy, *n* (%)	1505 (30.1)	1226 (36.4)	279 (19.4)	0.001
PCa family history, *n* (%)	352 (7.0)	246 (6.9)	106 (7.4)	0.001
Prostate volume, mL (IQR)	55 (40–76)	58 (42–80)	47 (35–77)	0.001
PI-RADS score, *n* (%)				
1	574 (11.5)	459 (12.9)	115 (8.0)	0.001
2	175 (3.5)	137 (3.8)	38 (2.6)	
3	1250 (25.0)	1051 (29.5)	199 (13.8)	
4	2015 (40.3)	1485 (41.6)	530 (36.8)	
5	991 (19.8)	434 (12.2)	557 (38.7)	
PCa detection, *n* (%)	2858 (57.1)	1778 (49.9)	1080 (75.8)	0.001
significant PCa	2097 (41.9)	1158 (32.5)	939 (65.3)	0.001
insignificant PCa	761 (15.2)	620 (17.4)	141 (9.8)	0.001

IQR = interquartile range; PSA = prostate-specific antigen; *n* = number; DRE = digital rectal examination; PCa = prostate cancer; PI-RADS = prostate imaging–reporting and data system; −DRE = negative DRE; +DRE = suspicious DRE.

**Table 2:** Multivariate analysis showing independent predictive variables for significant PCa detection.

Predictive Variable	Odd Ratio (95% CI)	*p*-Value
Age, Ref. year	1.063 (1.053–1.073)	0.001
Serum PSA, Ref. ng/mL	1.031 (1.023–1.039)	0.001
DRE, Ref. normal	2.198 (1.876–2.576)	0.001
Type of biopsy, Ref. initial	0.706 (0.605–0.825)	0.001
PCa family history, Ref. no	1.556 (1.190–2.035)	0.001
Prostate volume, Ref. mL	0.978 (0.975–0.980)	0.001
PI-RADS score, Ref. 1	2.586 (2.383–2.806)	0.001

CI = confidence interval; PSA = prostate-specific antigen; DRE = digital rectal examination; PCa = prostate cancer; PI-RADS = prostate imaging–reporting and data system.

## Data Availability

Data are available upon request to the corresponding author.

## Abbreviations

BCN-PM	Barcelona-Predictive Model
CI	Confidence Interval
DRE	Digital Rectal Examination
MRI	Magnetic Resonance Imaging
OR	Odds Ratio
PCa	Prostate Cancer
PI-RADS	Prostate Imaging-Reporting and Data System
RR	Relative Risk
